# A Novel Mix of Polyphenols and Micronutrients Reduces Adipogenesis and Promotes White Adipose Tissue Browning via UCP1 Expression and AMPK Activation

**DOI:** 10.3390/cells12050714

**Published:** 2023-02-24

**Authors:** Francesca Pacifici, Gina Malatesta, Caterina Mammi, Donatella Pastore, Vincenzo Marzolla, Camillo Ricordi, Francesca Chiereghin, Marco Infante, Giulia Donadel, Francesco Curcio, Annalisa Noce, Valentina Rovella, Davide Lauro, Manfredi Tesauro, Nicola Di Daniele, Enrico Garaci, Massimiliano Caprio, David Della-Morte

**Affiliations:** 1 Department of Systems Medicine, University of Rome “Tor Vergata”, 00133 Rome, Italy; 2 Laboratory of Cardiovascular Endocrinology, IRCCS San Raffaele, 00166 Rome, Italy; 3 Department of Human Sciences and Quality of Life Promotion, San Raffaele University, 00166 Rome, Italy; 4 Cell Transplant Center, Diabetes Research Institute, University of Miami Miller School of Medicine, Miami, FL 33136, USA; 5 Section of Diabetology, UniCamillus, Saint Camillus International University of Health Sciences, Via di Sant’Alessandro 8, 00131 Rome, Italy; 6 Department of Clinical Sciences and Translational Medicine, University of Rome Tor Vergata, 00133 Rome, Italy; 7 Covid Internal Medicine Unit, Department of Translational Medical Sciences, AOU Federico II, University of Naples Federico II, Via S. Pansini, 5, 80131 Naples, Italy; 8 UOC of Internal Medicine-Center of Hypertension and Nephrology Unit, Department of Systems Medicine, University of Rome Tor Vergata, Via Montpellier 1, 00133, Rome, Italy; 9 Department of Neurology, Evelyn F. McKnight Brain Institute, Miller School of Medicine, University of Miami, Miami, FL 33136, USA; 10 Interdisciplinary Center for Advanced Studies on Lab-on-Chip and Organ-on-Chip Applications (ICLOC), University of Rome Tor Vergata, 00133 Rome, Italy

**Keywords:** polyphenols, browning, adipogenesis, mitotic clonal expansion, AMPK, UCP1

## Abstract

**Background**: Obesity is a pandemic disease characterized by excessive severe body comorbidities. Reduction in fat accumulation represents a mechanism of prevention, and the replacement of white adipose tissue (WAT) with brown adipose tissue (BAT) has been proposed as one promising strategy against obesity. In the present study, we sought to investigate the ability of a natural mixture of polyphenols and micronutrients (A5^+^) to counteract white adipogenesis by promoting WAT browning. **Methods**: For this study, we employed a murine 3T3-L1 fibroblast cell line treated with A5^+^, or DMSO as control, during the differentiation in mature adipocytes for 10 days. Cell cycle analysis was performed using propidium iodide staining and cytofluorimetric analysis. Intracellular lipid contents were detected by Oil Red O staining. Inflammation Array, along with qRT-PCR and Western Blot analyses, served to measure the expression of the analyzed markers, such as pro-inflammatory cytokines. **Results**: A5^+^ administration significantly reduced lipids’ accumulation in adipocytes when compared to control cells (*p* < 0.005). Similarly, A5^+^ inhibited cellular proliferation during the mitotic clonal expansion (MCE), the most relevant stage in adipocytes differentiation (*p* < 0.0001). We also found that A5^+^ significantly reduced the release of pro-inflammatory cytokines, such as IL-6 and Leptin (*p* < 0.005), and promoted fat browning and fatty acid oxidation through increasing expression levels of genes related to BAT, such as UCP1 (*p* < 0.05). This thermogenic process is mediated via AMPK-ATGL pathway activation. **Conclusion**: Overall, these results demonstrated that the synergistic effect of compounds contained in A5^+^ may be able to counteract adipogenesis and then obesity by inducing fat browning.

## 1. Introduction

Obesity is a pandemic health problem [1]. In 2016, the World Health Organization (WHO) estimated that 650 million adults, 340 million adolescents and 39 million children were affected by obesity, and these numbers are growing fast [2]. This condition has been worsened by increased junk food consumption, highly enriched with sugar and fat, that contributes to the development of visceral adiposity, which is strongly associated with cardiovascular diseases (CVD) [3]. Visceral adiposity is primarily composed of white adipose tissue (WAT) and is the main type of adipose tissue serving as energy storage. WAT also acts as an endocrine organ, secreting several pro-inflammatory cytokines, such as tumor necrosis factor (TNF)-α, Interleukin (IL)-6, and leptin, among others [4]. In a state of obesity, the significant increase in WAT and in cytokine levels led to the onset of a pro-inflammatory state typical of this pathological condition and its related disorders (insulin resistance, diabetes mellitus, and CVD). 

Recently, it has been proposed that WAT transdifferentiation into brown adipose tissue (BAT), a phenomenon known as browning, may be a novel approach to counteract obesity [5]. BAT activation enhances energy expenditure and promotes a negative energy balance reducing weight gain in animal models [6,7]. BAT uncouples fatty acid oxidation from adenosine triphosphate (ATP) production, dissipating energy as heat [8]. This beneficial process is primarily mediated by AMP-activated protein kinase (AMPK) that, when triggered by specific impulses, such as cold and/or fasting, induces phosphorylation and activation of adipose triglyceride lipase (ATGL), leading to an increase in lipolysis and fatty acids (FA) release [7,9]. These FA, in turn, bind to the uncoupling protein 1 (UCP1), a protein located in the inner mitochondrial membrane, promoting the dissipation of an electrochemical gradient as heat [9]. 

Based on these known mechanisms, several pharmacological and nutritional approaches have been proposed to counteract obesity and fat accumulation [10]. Among nutritional compounds, polyphenols showed a significant anti-obesity effect by regulating lipid metabolism [11]. Resveratrol, the most studied among polyphenols, promotes BAT metabolism by increasing expression of UCP1 in rodents [12]. However, the major limitation in the clinical application of polyphenols, especially resveratrol, is their low bioavailability [13]. To avoid this problem, several resveratrol derivatives with enhanced bioavailability have been proposed and investigated, such as the glycosylated derivate polydatin and the methoxylated derivative pterostilbene [14]. Chronic pterostilbene administration in mice fed with a high fat diet has already been reported to improve lipid metabolism and to promote expression of UCP1 and other factors related to BAT [15]. Recently, we demonstrated that a novel mix of polyphenols and micronutrients, called A5^+^, was able to protect against inflammation by reducing cytokines-mediated processes in different in vitro experimental models [16,17].

Based on these findings, the present study aimed to evaluate the effects of A5^+^ in counteracting adipogenesis by promoting WAT browning in a model of 3T3-L1 murine fibroblasts. 

## 2. Materials and Methods

### 2.1. Cell Culture, Differentiation and Treatments

A murine 3T3-L1 fibroblast cell line was provided by Prof. Massimiliano Caprio (San Raffaele Open University) and cultured in Dulbecco’s Modified Eagle’s Medium (DMEM, 4.5 g/L glucose) (Gibco, Thermo Fisher Scientific, Waltham, MA, USA), supplemented with 10% Fetal Calf Serum and 1% penicillin/streptomycin (Gibco, Thermo Fisher Scientific, Waltham, MA, USA) at 37 °C in a humidified, 5% CO_2_ atmosphere. 

To induce differentiation, as previously reported [18], cells were seeded at the desired concentration in the culture medium. When they reached confluence, the medium was changed. The new differentiation medium was composed of DMEM 4.5 g/L glucose supplemented with 10% Fetal Bovine Serum (FBS, Corning, NY, USA), 1% penicillin/streptomycin, 1 µg/mL insulin, 0.5 mM isobutylmethylxanthine (IBMX), and 1 µM dexamethasone, 50 µM A5^+^ (SirtLIfe srl, Rome), or DMSO (for control cells) (Sigma Aldrich, Saint Louis, MO, USA) for 2 days. On day 2, the differentiation medium was replaced with DMEM (4.5 g/L glucose) containing 10% FBS, 1 µg/mL insulin, and 50 µM A5^+^, or DMSO (for control cells), until day 10. The medium was changed every 2 days until day 10.

A5^+^ is composed of ellagic acid (20%), polydatin (98%), pterostilbene (20%), and honokiol (20%), mixed with recommended doses of zinc, selenium, and chromium. It is dissolved in DMSO at 1 mg/mL, as reported by Pacifici et al. [17].

### 2.2. Oil Red O Staining

Oil Red O staining was performed to quantify the intracellular lipid content as previously described [18]. Briefly, 1 × 10^5^ cells were seeded in a 6-multiwell plate and differentiated as reported in Section 2.1. Then, the cells were washed and fixed with 4% formalin (Sigma Aldrich, Saint Louis, MO, USA). Subsequently, the cells were incubated with 60% isopropanol (Sigma Aldrich, Saint Louis, MO, USA) and then stained with Oil Red O solution (0.5 g/L, Sigma Aldrich, Saint Louis, MO, USA). The dye solution maintained by the cells was dissolved in pure isopropanol and quantified at 490 nm by using the Multiskan FC microplate reader (Thermo Fisher Scientific, Waltham, MA, USA).

### 2.3. Proliferation Assay

For cell proliferation, 2 × 10^4^ cells were plated in a 24-multiwell plate and differentiated as previously reported. At time 0 and at 48 h, the cells were detached using trypsin solution 0.05% (Gibco, Thermo Fisher Scientific, Waltham, MA, USA), then they were centrifuged and the pellet was resuspended in culture medium. Then, 10 µL o cell resuspension was added to 10 µL of trypan blue (Sigma Aldrich, Saint Louis, MO, USA) and analyzed with a Countess Automated Cell Counter (Thermo Fisher Scientific, Waltham, MA, USA).

### 2.4. Cell Cycle Analysis

Cell cycle analysis was performed using Propidium Iodide staining as reported in Pacifici et al. [17]. Briefly, the cells were seeded at 1 × 10^5^ in a 6-multiwell plate and differentiated as reported in Section 2.1. Then, both the supernatants and cells were collected in a FACS collection tube and centrifuged at 1600 rpm for 5 min. Subsequently, the supernatant was discarded, and the pellet was fixed with 70% ethanol for 45 min [19]. Finally, the cells were washed with PBS, stained with PI solution, and analyzed using cytofluorimetric analysis.

### 2.5. Inflammatory Array

Cytokines profile was analyzed in the supernatants of differentiated cells using the Mouse Inflammation Array C1 (Ray-Biotech, Inc., Norcross, GA, USA), as previously reported [17]. Briefly, the cells were treated as described in Section 2.1; at day 10, the supernatants were collected, centrifuged to remove cell debris, and used for the assay. Membranes with 40 spotted cytokine antibodies were blocked with the supplied blocking buffer and then incubated overnight at +4 °C with the supernatants. The next day, the membranes were washed and incubated overnight at +4 °C with a biotinylated antibody cocktail. The next day, the membranes were washed, and HRP-Streptavidin solution was added over night at +4 °C. The following day, the membranes were washed and detected by chemiluminescence. The membranes map is reported in Table 1.

### 2.6. Gene Expression Analysis

For gene expression analysis, total RNA was isolated by using TRIzol reagent (Invitrogen, Thermo Fisher Scientific, Waltham, MA, USA) according to the manufacturer’s protocol. Then, 2.5 µg of total RNA was reverse transcribed into cDNA by using the High-Capacity cDNA Archive Kit (Invitrogen, Thermo Fisher Scientific, Waltham, MA, USA). qRT-PCR was performed using the ABI Prism 7500 instrument (Applied Biosystem, Thermo Fisher Scientific, Waltham, MA, USA). cDNA amplification was assessed using a specific primer reported by Marzolla et al. [20] (UCP1, Adbr3, Cidea, DIO2, Cpt1beta, Cpt2, Crat, ACADM, ACADL, Hadha, Aco2, Idh3a Sdhac, Cs), and PowerUp SYBR green dye (Invitrogen, Thermo Fisher Scientific, Waltham, MA, USA) according to the manufacturer’s protocol. All samples were normalized using TATA-box binding protein (TBP) as an internal control; the relative quantification was calculated using the comparative ΔΔCT method, and the values were expressed as 2^−ΔΔCT^.

### 2.7. Western Blot Analysis

The 3T3-L1 cell pellets were lysed at 4 °C in an HNTG lysis buffer (1% Triton X-100, 50 mM HEPES, 10%glycerol, 150 mM NaCl, 1% sodium deoxycholate) supplemented with Phosphatase Inhibitor Cocktail 2 and 3 (Sigma Aldrich, Milan, Italy) and protease inhibitor cocktail (Sigma Aldrich, Milan, Italy). A clear supernatant was obtained by centrifugation of lysates at 13,000× *g* for 15 min at 4 °C. Protein concentration was determined using a BCA protein assay kit (Pierce; Thermo Fisher Scientific, Milan, Italy). Protein samples were subjected to sodium dodecylsulfate polyacrilamide gel electrophoresis (SDS-PAGE) using Miniprotean precast gels (BioRad; Segrate, Italy) and electroblotted onto nitrocellulose membranes (Bio-Rad, Segrate, Italy). Membranes were blocked for 1 h at room temperature (RT) with 5% non-fat milk in Tris-Buffered Saline with 0.05% Tween 20 (TBS-T). Incubation with primary specific antibodies was performed in the blocking solution (5% milk or bovine serum albumin in TBS-T) overnight at 4 °C and horseradish peroxidase-conjugated secondary antibodies (in blocking solution) for 1 h at RT. We used antibodies against AMPK-α 1:1000 (Cell Signaling, Danvers, MA, USA), phospho-AMPK-α (Thr172) 1:1000 (Cell Signaling, Danvers, MA), ATGL 1:1000 (Cell Signaling, Danvers, MA), phospho-ATGL (Ser406) 1:1000 (Abcam Cambridge, MA, USA), and UCP1 1:1000 (Abcam Cambridge, MA, USA). The appropriate secondary horseradish peroxidase-conjugated antibodies from Jackson Immunoresearch were used in the blocking solution (1:5000). Immunoreactive bands were visualized by Luminata Forte Western Chemiluminescent HRP substrate (Millipore (Merk); Milan, Italy) using an ImageQuant LAS 4000 (GE Healthcare). Equal samples loading was confirmed using GAPDH 1:30,000 (Sigma Aldrich, Milan, Italy) and bands quantified by densitometry using the ImageQuant TL software from GE Healthcare Life Sciences.

### 2.8. Statistical Analysis

All data were analyzed using GraphPad Prism 9 (La Jolla, CA, USA). An unpaired two-tailed Student’s test was used for statistical analysis and significance. All data were expressed as mean ± SEM. Values of *p* < 0.05 were considered statistically significant.

## 3. Results

### 3.1. A5^+^ Blunts Intracellular Lipid Accumulation

**Figure 1:**
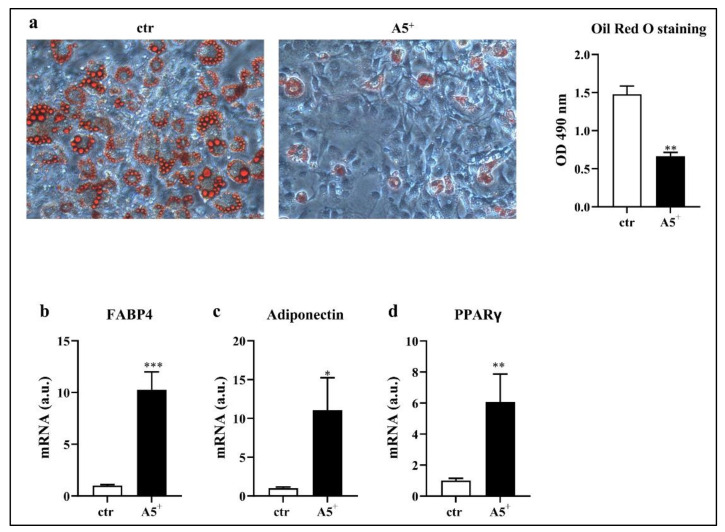
Oil Red O staining in 3T3-L1 adipocytes. Cells were seeded at a density of 1 × 10^5^ cells/well in a 6-well plate and differentiated with or without A5^+^. (**a**) Cells were stained with Oil Red O and lipid droplets were visualized in optical microscopy (20× magnification) and quantified by measuring absorbance. (**b**–**d**) Bar graphs illustrating the most relevant adipogenesis-related genes modulated by A5^+^. Results are expressed as the mean ± SEM. * *p* < 0.05, ** *p* < 0,005, *** *p* < 0.001. Graphs illustrate three different experiments conducted separately. FAB4: Fatty acid-binding protein 4; PPARγ (peroxisome proliferator-activated receptor gamma).

In order to test whether A5^+^ was able to reduce intracellular lipid accumulation, we induced 3T3-L1 differentiation into a mature adipocyte phenotype. Then, we stained the differentiated cells with an Oil Red O solution that recognized triglycerides and lipids. As reported in Figure 1, A5^+^ administration significantly reduced lipid accumulation when compared to control cells, as confirmed by the Oil Red O absorbance at 490 nm (*p* < 0.005). These results indicated a direct effect of this compound on the mechanisms associated with fat storage. To further validate a reduction in adipogenesis, we also analyzed the mRNA expression of some adipogenic factors (Figure 1, Panels b–d). Accordingly, we observed a significant increase in FAB4 (*p* < 0.001) and adiponectin expression (*p* < 0.05) in the A5^+^-treated cells. Moreover, PPARγ levels were increased following A5^+^ administration, in agreement with its ability to promote adipogenesis in both white in brown adipose tissue, and to boost the brown-fat characteristics in white adipose tissue [21]. Taken together, these data suggest an involvement of A5^+^ in reducing white adipocytes maturation.

### 3.2. A5^+^ Inhibits Cell Proliferation by Arresting the Cell Cycle in G2-M Phase

**Figure 2:**
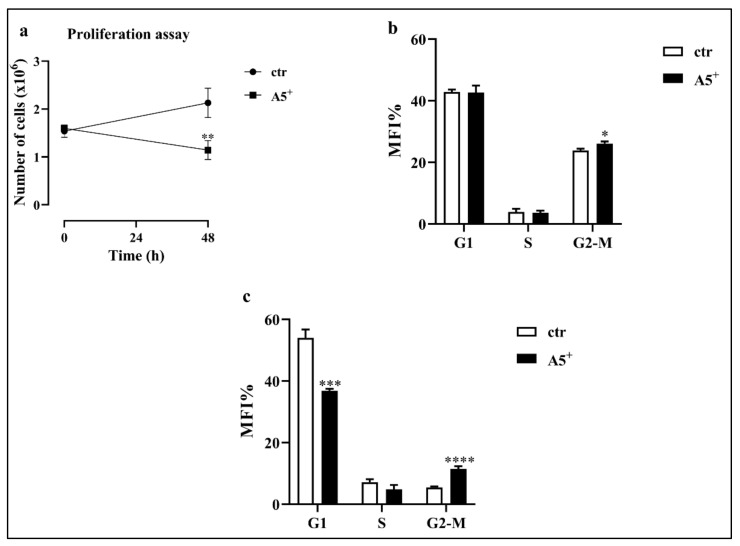
Cell proliferation and cell cycle analysis. (**a**) Cell proliferation during MCE; (**b**) Cell cycle analysis during MCE; (**c**) Cell cycle analysis at day 10. Results are expressed as the mean ± SEM. * *p* < 0.05, ** *p* < 0.005, *** *p* < 0.001, **** *p* < 0.0001. Graphs illustrate three different experiments conducted separately.

Mitotic clonal expansion (MCE) is one of the most relevant stages in adipocytes differentiation. MCE is the moment where the cells reentered the cell cycle and promoted the transcription of several genes involved in 3T3-L1 adipocytes differentiation [22]. Based on the importance of MCE, we tested whether A5^+^ could act at this stage by reducing cell proliferation and thus, the differentiation driving force. Cells were plated at 1 × 10^5^ cells/well in a 6-multiweel plate and differentiation was induced as previously reported. Then, at day 2, cell number and cell cycle were assessed. As expected, while physiological proliferation occurred in control cells, A5^+^ administration significantly reduced cell proliferation (*p* < 0.005) (Figure 2, Panel a). We also evaluated the cycle to confirm the cell growth arrest mediated by the selected compound. As reported in Figure 2, Panel b, cells treated with A5^+^ showed a cell cycle arrest in G2-M phase compared to control cells (*p* < 0.05). These results were further confirmed by the G2-M cell cycle arrest observed during the follow-up of this process with a peak at day 10 (*p* < 0.0001) (Figure 2, Panel c).

### 3.3. A5^+^ Administration Blunts Inflammatory Cytokines Release in Adipocytes

**Figure 3:**
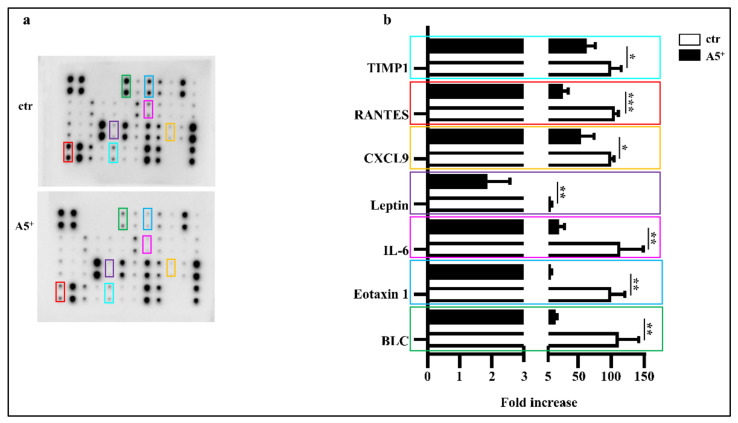
A5^+^ reduced adipocytes induced inflammation. (**a**) Representative blot assay reporting all cytokines evaluated; (**b**) Bar graph illustrating the most relevant cytokines modulated by A5^+^ administration. Data are reported as mean ± SEM (*n* = 4). (* *p* < 0.05; ** *p* < 0.005; *** *p* < 0.001). ctr: control; BLC: B lymphocyte chemoattractant; IL-6 Interlukin-6; CXCL9: chemokine (C-X-C motif) ligand 9; RANTES: Regulated upon Activation, Normal T Cell Expressed and Secreted; TIMP1: Tissue inhibitor matrix metalloproteinase 1.

It is well known that mature adipocytes secrete several pro-inflammatory cytokines, thereby contributing to systemic inflammation and complications in obese subjects [23]. In order to evaluate whether this novel compound may impact on inflammation, we tested the secretion levels of several cytokines directly involved in adipocytes maturation and lipid accumulation in a differentiated mature 3T3-L1 adipocytes medium. As shown in Figure 3, A5^+^ administration significantly reduced the release of BLC, Eotaxin 1, IL-6, Leptin (*p* < 0.005), the chemokin CXCL9 (*p* < 0.05), RANTES (*p* < 0.001), and TIMP1 (*p* < 0.05) when compared to control cells. These data further highlight the relevant anti-inflammatory effect of polyphenols in general, and A5^+^ in particular. These findings are also in agreement with our previous data [17].

### 3.4. A5^+^ Promotes Fat Browning

**Figure 4:**
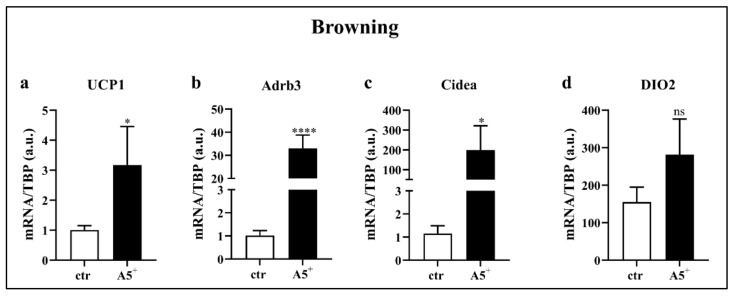
A5^+^ promotes browning related genes. Bar graph illustrating the most relevant browning-related genes modulated by A5^+^ administration: (**a**) UCP1, (**b**) Adrb3, (**c**) Cidea, (**d**) DIO2. Data are reported as mean ± SEM (*n* = 4). (* *p* < 0.05; **** *p* < 0.0001). ctr: control; UCP1: uncoupling protein 1; Adrb3: β3-adrenergic receptor; DIO2: Deiodinase, iodothyronine, type II. ns: not significant.

Recently, a novel strategy to counteract obesity has been reported: it is based on the increase in activity and/or amount of brown adipose tissue (BAT), which, as opposed to WAT, dissipates energy by generating heat and leading to a negative energy balance and weight loss [6]. Based on our previous results, we tested whether reduction in lipid content after A5^+^ treatment may be attributed to fat browning. Therefore, we differentiated cells and isolated RNA to evaluate gene expression levels of important genes related to BAT. As reported in Figure 4, cells treated with A5^+^ displayed significantly increased levels of UCP1 (*p* < 0.05), Adrb3 (*p* < 0.0001), and Cidea (*p* < 0.05). A positive but non-significant trend was also shown for DIO2. These data demonstrated that this natural compound was able to promote fat browning, suggesting a potential role in blunting fat accumulation and obesity by triggering the switch from WAT to BAT.

### 3.5. A5^+^ Regulates Lipid Metabolism

**Figure 5:**
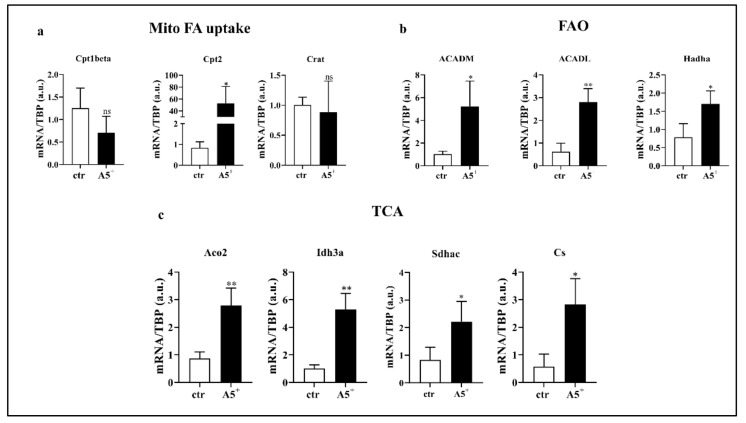
A5^+^-induced lipid metabolism. Bar graph illustrating the most relevant lipid metabolism-related genes modulated by A5^+^ administration: (**a**) Genes related to mitochondrial fatty acids (FA) uptake, (**b**) Genes involved in fatty acid oxidation (FAO), (**c**) Genes regulating tricarboxylic acid (TCA) cycle. Data are reported as mean ± SEM (*n* = 4). (* *p* < 0.05; ** *p* < 0.005; ns: not significant). ctr: control; Cpt1beta: Carnitine palmitoyltransferase I beta; Cpt2: Carnitine palmitoyltransferase II; Crat: Carnitine acetyltransferase; ACADM: acyl-Coenzyme A dehydrogenase, C-4 to C-12 straight chain; ACADL: Acyl-CoA dehydrogenase, long chain; Hadha: Hydroxyacyl-CoA dehydrogenase/3-ketoacyl-CoA thiolase/enoyl-CoA hydratase, alpha subunit; Aco2: aconitase 2; Idh3a: Isocitrate dehydrogenase subunit alpha; Sdhac: Succinate dehydrogenase complex subunit C; Cs: Citrate synthase; TBP: TATA box binding protein.

Fatty acid (FA) oxidation is essential to induce UCP1 expression and, thus, to maintain and develop fat browning [24]. Based on our results showing the up-regulation of browning-related genes, we decided to analyze expression levels of genes involved in FA oxidation (Figure 5). As expected, genes involved in mitochondrial FA uptake, in particular Cpt2, significantly increased in A5^+^-treated cells when compared to control (ctr) cells (*p* < 0.05) (Figure 5, Panel a). Moreover, following A5^+^ administration, all analyzed components linked to FA oxidation increased when compared to ctr cells (ACADM: *p* < 0.05; ACADL: *p* < 0.005; Hadha: *p* < 0.05) (Figure 5, Panel b). The Acetyl-Coa derived from FA, metabolized by FAO, enters the TCA cycle to produce the most relevant cofactors essential for mitochondrial respiration [25]. According to the previously shown results genes involved in the TCA cycle were upregulated after treatment (Aco2 and Idh3a: *p* < 0.005; Sdhac and Cs: *p* < 0.05) (Figure 5, Panel c). Taken together, these data suggest that A5^+^ regulates brown fat thermogenesis.

### 3.6. A5^+^ Regulates Cellular Lipid Metabolism in 3T3-L1 via AMPK-ATGL Pathway

**Figure 6:**
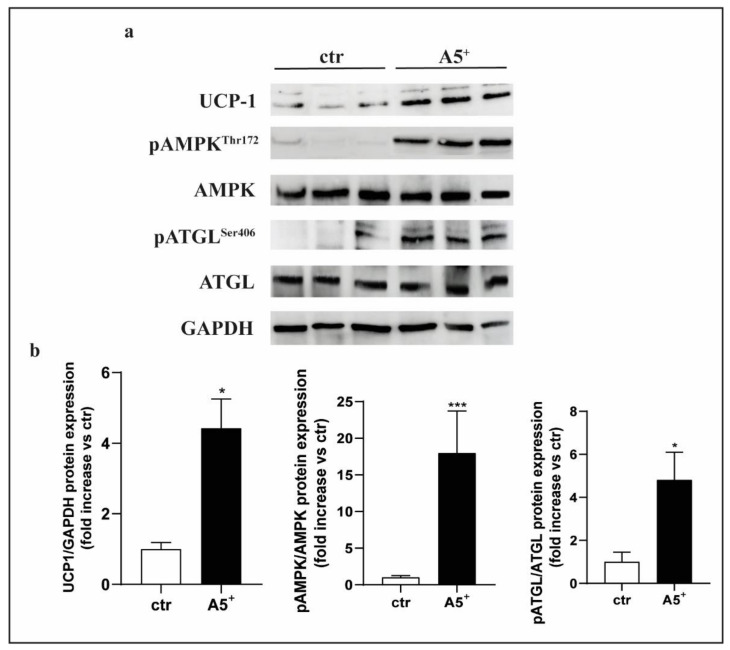
A5^+^ determined activation of AMPK-ATGL pathway in 3T3-L1 adipocytes. (**a**) Representative immunoblots of UCP1, AMPK, and ATGL activation analysis (*n* = 6), (**b**) distribution graphs of the densitometric scanning analyses performed by ImageQuant TL software by using GAPDH as loading control. Phosphorylated forms were normalized in comparison with their total forms. * *p* < 0.05; *** *p* < 0.001.

The observation that A5^+^ treatment increases the expression of thermogenesis-related markers prompted us to investigate the molecular mechanisms underlying the browning of 3T3-L1 adipocytes. 3T3-L1 pre-adipocytes were differentiated, in complete medium, in the presence or absence of A5^+^ for 10 days. A5^+^ effects on 3T3-L1 cells were assessed using western blot analysis of UCP-1 protein expression in terminally differentiated 3T3-L1 cells (day 10). A significant increase of UCP-1 protein expression was observed in A5^+^-treated 3T3-L1 cells when compared with control cells (*p* < 0.05) (Figure 6, Panel b). Given the well-known role of AMP-activated protein kinase (AMPK) as a sensor of intracellular energy state by regulating FA metabolism and thermogenesis in adipose tissue [26], we investigated whether A5^+^ was able to activate AMPK. We observed that A5^+^ administration induced a significant increase of AMPK-α phosphorylation at threonine-172 (Thr172) at day 10 of 3T3-L1 cell differentiation, indicating its capacity to induce AMPK activation (*p* < 0.001) (Figure 6, Panel b). Adipose triglyceride lipase (ATGL) can be phosphorylated at serine-406 (Ser406) by AMPK to increase its catalytic activity and, in turn, lipolysis in adipocytes [27].Therefore, we examined ATGL phosphorylation at Ser406 in A5^+^-treated 3T3-L1 cells and observed that it was significantly increased in A5^+^-treated cells when compared to control cells (*p* < 0.05) (Figure 6, Panel b).

## 4. Discussion

In the present study, by using a model of a 3T3-L1 fibroblast cell line differentiated into mature adipocytes, we reported, for the first time, that a mix of polyphenols and micronutrients (A5^+^) may be useful in preventing obesity and its related complications. A5^+^ administration reduced the accumulation of intracellular lipids and inhibited adipocytes differentiation during MCE, therefore blunting fat accumulation. Moreover, as reported in our previous studies [16,17], A5^+^ significantly reduced the release of pro-inflammatory cytokines, including leptin. All these beneficial properties of A5^+^ were primarily linked to its ability to triggering fat browning, or rather switching white adipose tissue to brown adipose tissue, as demonstrated by an increase in the genes linked with this mechanism and with fatty acid oxidation. At a molecular level, overexpression of UCP1 and the activation of AMPK represented the main thermogenic pathways involved.

Recently, we showed that A5^+^ significantly blunted inflammation in an in vitro model of Parkinson’s disease [17]. This relevant effect was explained, at least in part, by the synergistic and integrative effect of its components that act in different phases of cellular rescue mechanisms against damage and/or cellular stress. Similarly, in obesity, where a low grade of inflammation plays a pivotal role [28], the components of A5^+^ may induce a preventive and protective effect. The efficacy of the different polyphenols against obesity has been already largely explored and reported [29]. We previously demonstrated that tyrosol, a major polyphenol found in extra virgin olive oil, inhibited adipogenesis by downregulating several adipogenic factors (leptin and aP2) and transcription factors (C/EBPα, PPARγ, SREBP1c, and Glut4) and by modulating the histone deacetylase sirtuin 1 [18]. A study using the same in vitro model of the present research, showed that phenolic acids, including ellagic acid, inhibited lipid accumulation throughout the whole process of adipogenesis differentiation [30]. However, in this study it was remarked that, despite the similar structure of these compounds, they show interactions with different targets when compared to those reported in the previous study; they also exert distinct effects in adipogenesis [30]. Moreover, polydatin, pterostilbene, and honokiol were not tested. Polydatin was shown to reduce body weight in high fat diet (HFD)-fed mice and to downregulate serum levels of triglyceride, low density lipoprotein (LDL), aspartate aminotransferase (AST), and alanine aminotransferase (ALT), and to upregulate high-density lipoprotein (HDL) [31]. In association with the loss of weight, polydatin also reduced levels of pro-inflammatory factors such as IL-6 [31]. On the other hand, pterostilbene significantly ameliorated free fatty acids (FFA)-induced lipid accumulation in HepG2 cells and activated FA β-oxidation to inhibit FA synthesis in HFD-fed mice via AMPK activation [32]. Again, honokiol supplementation promoted the browning of WAT by upregulation of UCP1 and AMPK expression in HFD mice [33]. All these findings are completely in line with the results of the present study and with our hypothesis of the concomitant and interactive effect of the A5^+^ compounds on the adipogenesis mechanisms. 

After A5^+^ treatment, we found a cell cycle arrest in the G2-M phase during adipogenesis, which may be the main cause of the following cascade effect, including reduction in cytokine cellular secretion. Among all pro-inflammatory factors, a significant decrease in leptin release was found. This may have an important consequence since leptin is a primary adipokine linked to mechanisms leading to obesity and its complications regulating body mass via negative feedback between adipose tissue and hypothalamus. [28,34]. In turn, the reduction of IL-6 and CXCL9, that increase the concentration of FFA [35], may drive the regulation of mitochondrial FA metabolism. 

The ultimate protective step of induced by A5^+^ is the promotion of fat browning. This is a complex process in which gut microbiota also plays an important role [36]. BAT includes several cells, such as pre-adipocytes, stem progenitor cells, and immune cells, and has anti-inflammatory action through the ability to dissipate energy in the form of heat, primarily mediated by UCP1 [8]. In obesity, BAT function is negatively affected by inflammatory mediators, such as high levels of cytokines. For this reason, anti-inflammatory supplementation, even natural, has already been proposed to preserve it [5]. Here we found that treatment with A5^+^ increases the expression of the main genes involved in fat browning and in FA oxidation. These processes control adipose tissue thermogenesis [8]. UCP1 generates a heat dissipating energy proton gradient from the electron transport chain in mitochondrial respiration [37]. The increase in cellular respiration has favorable effects on other cellular pathways such as AMPK-ATGL, which, in turn, are pivotal to activate central and peripheral beneficial effects of BAT [9]. Here, we demonstrated either an increase of UCP1 and AMPK-ATGL expression after A5^+^ treatment. Interestingly, AMPK has already been shown to be positively modulated by other polyphenols, such as resveratrol [9]. 

The beneficial effect of minerals dissolved in A5^+^ (zinc, selenium, and chromium) against obesity has been largely demonstrated. Recently, the levels of these elements were found to be significantly reduced when measured in blood serum, hair, and urine of obese adult patients, demonstrating their predictive role in obesity and the helpful impact of their adequate replacement therapy [38]. 

## 5. Conclusions

**Figure 7:**
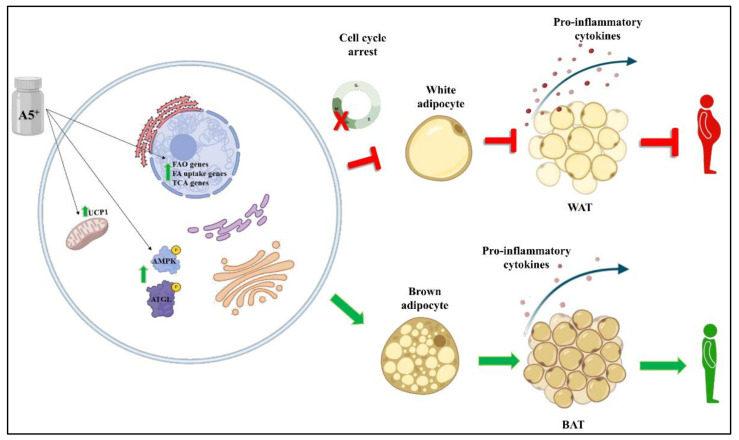
Schematic representation of A5^+^ effects on adipogenesis and browning. A5^+^ administration increased the expression levels of several genes involved in FAO, FA uptake and TCA. Moreover, it also promoted the activation of the AMPK-ATGL pathway and increased the expression levels of UCP1, leading to BAT generation and reducing the pro-inflammatory state typical of obesity and white adipogenesis. Created by Bio-Render.com.

In conclusion, in the present article we found that a natural product composed of highly bioavailable polyphenols and minerals may help in preventing some cellular processes associated with obesity, primarily by reducing cellular lipid accumulation and by increasing fat browning through enhancement of mitochondrial respiration and fatty acid oxidation (Figure 7). Further studies in this important field are necessary to understand how to counteract this pandemic disease.

## Figures and Tables

**Table 1:** Membrane cytokines array map.

POS	POS	NEG	NEG	Blank	BLC	CD30L	Eotaxin	Eotaxin-2	Fas L	Fractalkine	GCSF
POS	POS	NEG	NEG	Blank	BLC	CD30L	Eotaxin	Eotaxin-2	Fas L	Fractalkine	GCSF
GM-CSF	IFNγ	IL-1α	IL-1β	IL-2	IL-3	IL-4	IL-6	IL-9	IL-10	IL-12 p40p70	IL-12 p70
GM-CSF	IFNγ	IL-1α	IL-1β	IL-2	IL-3	IL-4	IL-6	IL-9	IL-10	IL-12 p40p70	IL-12 p70
IL-13	IL-17	I-TAC	KC	Leptin	LIX	Lymphotactin	MCP-1	MCSF	MIG	MIP-1α	MIP-1γ
IL-13	IL-17	I-TAC	KC	Leptin	LIX	Lymphotactin	MCP-1	MCSF	MIG	MIP-1α	MIP-1γ
RANTES	SDF-1	TCA-3	TECK	TIMP-1	TIMP-2	TNF-α	sTNF RI	sTNF RII	Blank	Blank	POS
RANTES	SDF-1	TCA-3	TECK	TIMP-1	TIMP-2	TNF-α	sTNF RI	sTNF RII	Blank	Blank	POS

## Data Availability

The data are contained within the article.
